# Electrochemical Biosensors for Rapid Detection of Foodborne *Salmonella*: A Critical Overview

**DOI:** 10.3390/s17081910

**Published:** 2017-08-18

**Authors:** Stefano Cinti, Giulia Volpe, Silvia Piermarini, Elisabetta Delibato, Giuseppe Palleschi

**Affiliations:** 1 Department of Chemical Science and Technology, University of Rome “Tor Vergata”, Via della Ricerca Scientifica 1, 00133 Rome, Italy; stefano.cinti@uniroma2.it (S.C.); silvia.piermarini@uniroma2.it (S.P.); giuseppe.palleschi@uniroma2.it (G.P.); 2 Department of Veterinary Public Health and Food Safety, Istituto Superiore di Sanità, Viale Regina Elena 299, 00161 Rome, Italy; elisabetta.delibato@iss.it

**Keywords:** *Salmonella*, electrochemical detection, immunosensors, genosensors, aptasensors, phagosensors, food analysis

## Abstract

*Salmonella* has represented the most common and primary cause of food poisoning in many countries for at least over 100 years. Its detection is still primarily based on traditional microbiological culture methods which are labor-intensive, extremely time consuming, and not suitable for testing a large number of samples. Accordingly, great efforts to develop rapid, sensitive and specific methods, easy to use, and suitable for multi-sample analysis, have been made and continue. Biosensor-based technology has all the potentialities to meet these requirements. In this paper, we review the features of the electrochemical immunosensors, genosensors, aptasensors and phagosensors developed in the last five years for *Salmonella* detection, focusing on the critical aspects of their application in food analysis.

## 1. Introduction

For more than a century, *Salmonella* has been recognized as the most frequently occurring pathogen in food affecting human’s health. Salmonellosis has traditionally been linked to the consumption of food products of animal origin (e.g., meat, milk, and eggs) but, more recently, an increasing number of outbreaks has been associated with contaminated fruits and fresh vegetables [1,2,3,4]. Infection symptoms, such as abdominal pains, fever, nausea, vomiting, diarrhea, dehydration, weakness, and loss of appetite, normally appear 12–72 h after ingestion of contaminated foods or beverages.

Due to the low infective dose of *Salmonella* and the high number of subjects that may be affected in a single outbreak, Commission for food safety regulation (EC) N° 2073/2005 has established that viable *Salmonella* cells must be absent in a defined amount of a given food product [5]. The routine method to detect *Salmonella* in food is the standard cultural method (EN/ISO 6579) which entails a non-selective pre-enrichment step followed by a selective enrichment (to enhance the number of *Salmonella* cells versus the competitor microorganisms), isolation on selective agar medium, bacterial identification by biochemical and serological tests, to confirm the suspect colonies grown on the selective agar. Although this method is very sensitive and inexpensive, it is labor-intensive, extremely time consuming (up to five days to obtain results), and not suitable for testing a large number of samples. To overcome these drawbacks, research is focusing on the development of rapid, sensitive, and specific methods, easy to use, and suitable for multi-sample analysis. Biosensors, with particular reference to the electrochemical immunosensors, genosensors, aptasensors and phagosensors, match the above-cited requirements. Some authors, working in the biosensor field, highlight the need to develop new methodologies able to detect a single *Salmonella* cell in a defined amount of food product, without the pre-enrichment step [6]. In this regard, we want to specify that the pre-enrichment phase is essential to allow the growth of viable cells, overcoming the inability of the emerging methodologies (such as polymerase chain reaction (PCR) and biological recognition element-based methods) to distinguish between living and dead cells. Therefore, in this context, it appears clear that the development of highly sensitive methods, to establish the presence/absence of *Salmonella* in food, should be targeted to detect a single viable cell, reducing the pre-enrichment phase as much as possible. Phagosensors could represent a valid alternative to avoid the pre-enrichment phase, but to date their effectiveness to reach a so low limit has not been proven. Moreover, as recently reported by Labib and co-workers, a highly specific aptasensor was developed to distinguish between viable and heat killed *S*. Typhimurium cells [7]. This aptasensor was able to detect 600 CFU/mL as the lowest concentration, therefore the pre-enrichment step is still required to allow the *Salmonella* reaching a detectable level.

It is important to stress that highly sensitive methods must also be very selective because *Salmonella* often represents a small fraction of a large population of non-target organisms (endogenous microflora) present in food samples [8]. Moreover, proteins, carbohydrates, fats, hormones, and other nutrients might affect the measurement [6].

Another important aspect that we like to emphasize is that, although biosensors show a great potential for *Salmonella* screening, most of them are only used for the detection of a single *Salmonella* serotype (i.e., *S*. Typhimurium), and their ability to reveal different serovars has not been demonstrated yet. Thus, the selection of a biological recognition element able to interact with the most common *Salmonella* serotypes, isolated from food, is of crucial importance.

This review describes the most recent (over the last five years) electrochemical immunosensors, genosensors, aptasensors and phagosensors for *Salmonella* detection, paying particular attention to those applied in food analysis.

Among several approaches, we have chosen to focus on the electrochemical-based mechanisms of transduction [9,10]. One of the major challenges and opportunities of the field relies on developing smart sensor platforms which are cost effective, efficient, easy to use, and capable of minimizing tasks at the end user stage. These sensor platforms, coupled with biological recognition elements such as enzymes, antibodies, DNA, aptamers, and others, are gaining a leader position in the production of analytical devices due to their operational simplicity and to its “blindness” towards colored/turbid solutions, which normally reduce the application of the colorimetric tests in real samples. Moreover, thanks to the recent development of nanomaterials (metallic nanoparticles, conductive polymers, carbonaceous materials, i.e., graphene, nanotubes, carbon black), electrochemical biosensors take advantage of the easy manipulation and the unique chemical-physical properties of these cutting-edge materials (i.e., conductivity and surface-to-volume ratio) to greatly improve the analytical performances [11,12,13,14,15,16,17]. In addition, screen-printed technology (also known as thick film technology) represents the most favorable strategy to develop biosensors suitable for on-site and rapid analysis [18,19,20,21]. The ability to be easily mass producible allows the use of screen-printed electrodes (SPEs) as one-shot sensors. Thanks to the high adaptability of SPEs (i.e., customizing shape, dimension, conductive-ink material, and substrate), it is possible to fabricate selective and finely calibrated SPE-based biosensors specific for target analytes. Moreover, their surface modification with nanomaterials greatly improves the analytical performances, such as sensitivity, selectivity, reproducibility, accuracy, etc. [22,23,24,25,26].

The four classes of electrochemical biosensors described for *Salmonella* screening, reviewed in this paper, can be divided in two main subclasses: label-based and label free. They are essentially based on the use of SPEs coupled with nano- and micro-sized materials, such as gold nanoparticles (GNPs), carbon nanotubes (CNTs), graphene (GR), magnetic particles (MBs), quantum dots (QDs) and conductive polymers, employed to modify the electrode surface and/or as labels to generate highly performing analytical tools.

For sake of clarity, within each subclass, we report some examples that employ the same nano- and micro-size materials (i.e., GNPs, MBs, QDs, etc.) to better compare their analytical performances.

## 2. Nano- and Micro-Sized Materials for Improving Detection

A plethora of nano/micro materials has been utilized to develop and improve analytical methods for *Salmonella* detection. However, these materials find different application depending on the specific assay format. Among these, GNPs can be used as electrode modifier, promoting the electron transfer (due to their excellent electroactivity and surface-to-volume ratio), or as redox probes exploiting their detection in acidic media (following a preliminary oxidative dissolution of gold atoms) [27,28]. Unlike GNPs, CNTs are only capable of amplifying the conductivity of the electrode improving the loading of the “real” sensing (bio)element [29,30]. Their high electrical conductivity, stability, and mechanical strength is particularly required in developing superior electrochemical platforms. Conductive polymers can be also employed due to their highly π-conjugated polymeric chains, which provide both electronic and charge carriers [31]. Moreover, as for the PPy, conductivity can be finely tuned by using different doping agents and varying the electropolymerization conditions: this can lead to develop surfaces that are capable to discriminate different classes of bacterial strains [32]. In addition, multiplexed electrochemical assays can be developed with the use of QDs due to their great advantage in differentiating the signal that depend exclusively by the metal associated with sulfur, i.e., CdS, CuS, and PbS (as for the GNPs, the metal atom is dissolved and electrochemically detected) [33,34,35]. Besides the electrochemical improvement, nano/micro-sized MBs find plenty of applications due to both their large surface area (useful to load recognition elements) and their magnetic guidance. MBs are easily recovered by means of an external magnetic source, offering great simplifications in both terms of target pre-concentration and interferences reduction [36,37].

## 3. Electrochemical Immunosensors for *Salmonella* Detection

Electrochemical immunosensors are affinity ligand biosensors based on solid-state devices in which immunochemical reactions occur on a transducer surface to generate an electrochemical signal. The concept of the immunosensor methodology is similar to the conventional ELISA (Enzyme-Linked Immunosorbent Assay), however, in contrast to this immunoassay, modern transducer technology allows the highly sensitive determination of the immuno complex (antibody–antigen) in different ways [38].

Label-based electrochemical immunosensors require a marker (label) attached to an antigen (Ag) or antibody (Ab) to achieve an electron transfer. During the readout, the amount of label is detected and it is assumed to correspond to the concentration of the target analyte. Most of the immunosensors for bacteria detection are based on a sandwich format, in which the target cell is captured between two specific antibodies (Ab immobilized on the electrode surface or other supports and Ab labeled with a marker). The marker may itself be electroactive or able to generate an electroactive product [39] directly on the transducer surface. When specified by the authors, the antibodies used to capture *Salmonella* cells mainly recognize oligosaccharides (such as O or core antigens) of the membrane lipopolysaccharide (LPS) and, less frequently, the outer membrane proteins (Omp_s_). Enzymes, GNPs, and QDs are the most commonly employed markers for *Salmonella* detection. Moreover, GNPs are often used to modify the working electrode surface.

As labeling a molecule with various agents might influence the efficiency of the binding event, and the yield of the molecule-label coupling reaction is highly variable [40,41], the use of label free electrochemical immunosensors has become increasingly popular over the years. Electrochemical impedance spectroscopy (EIS) is the most widely used detection technique that normally requires the addition of an external redox probe (i.e., [Fe(CN)_6_]^3−/4−^, as marker. The electron transfer from the marker to the electrode is affected by the binding event which occurs on the electrode surface.

### 3.1. Label-Based Electrochemical Immunosensors

#### 3.1.1. GNPs

Xiang [42] and Fei [43] developed label-based electrochemical immunosensors modifying the electrode surface with GNPs. In particular, Xiang and colleagues proposed an ultrasensitive electrochemical immunosensor for *Salmonella* detection. GNPs were dispersed in chitosan hydrogel and used to modify a glassy carbon electrode, forming a composite film (GNPs/Chi). The biopolymer chitosan was oxidized (by applying an anodic potential to the electrode) and used as a platform to immobilize anti-*Salmonella* capture antibodies (Ab_1_). After incubation of the modified electrode in *Salmonella* suspension and horseradish peroxidase (HRP) conjugated secondary antibody (Ab_2_) solution, a sandwich electrochemical immunosensor was constructed. A wide linear range, from 10 to 10^5^ CFU/mL, and a low detection limit (LOD = 5 CFU/mL) were obtained using Differential Pulse Voltammetry (DPV). The authors claim that the high sensitivity of the immunosensor may be attributed to the following reasons: (i) the high surface area of GNPs, which substantially increases the loading of Ab_1_; (ii) the good biocompatibility of GNPs decorated chitosan, which provides a favorable environment to maintain the active configuration of the immunocomplex; and (iii) the very good conductivity of GNPs/Chi, which facilitates the electron transfer. The analysis time of this immunosensors takes about 4 h.

In our opinion, the two major drawbacks of this immunosensor are the following: (i) the working electrode is a bulk glassy carbon electrode that must be carefully cleaned before the deposition of the composite film, thus the entire procedure, for the fabrication of the immunosensor, has to be repeated for each *Salmonella* measurement; and (ii) its effectiveness in the detection of *Salmonella* in food samples has not been faced.

A sandwich electrochemical immunosensor for *Salmonella* Pullorum and Gallinarum was set up by Fei et al. [43]. GNPs were electrodeposited onto the surface of a carbon-based SPE for capturing antibodies and enhancing signals. Moreover, to generate a favorable microenvironment for the antibody (in terms of activity and stability), an ionic liquid was employed to modify the electrode surface. The immunosensor fabrication takes about 13 h but the authors have demonstrated its stability at 4 °C until use. After interaction between the capture antibody and *Salmonella* (40 min), a second antibody, labeled with HRP, was added and incubated for 40 min to form the sandwich complex. Hydrogen peroxide and thionine (reduced form) were used as HRP substrates and the enzymatic product (thionine oxidized form) was detected via Cyclic Voltammetry (CV), measuring the reduction peak. This immunosensor was capable of detecting *S*. Pullorum and Gallinarum in the concentration range 10^4^–10^9^ CFU/mL with a LOD of 3 × 10^3^ CFU/mL.

To verify the applicability of the immunosensor in food analysis, negative eggs and chicken meat samples were used. Some of these samples were randomly spiked with a proper dose of *S*. Pullorum and Gallinarum, and mixed with other negative samples. After an appropriate treatment, not specified by the authors, all samples were analyzed using both the immunosensor and the standard culture method. A good agreement between the two methods was obtained (especially in terms of true positive rate). Since this paper does not include either the sample treatment or the use of a pre-enrichment phase, we suppose that the positive samples were prepared to contain high *Salmonella* concentrations (≥3 × 10^3^ CFU/mL after treatment). This immunosensor, based on the use of SPE, is simpler, faster and more convenient to use than the previous one [42], but it displayed a lower sensitivity.

#### 3.1.2. MBs

Several biosensors, belonging to the label-based electrochemical immunosensors, use the immunomagnetic separation with antibody-modified magnetic particles for *Salmonella* detection.

Afonso and co-workers [44] employed MBs as support of a sandwich immunological complex in which GNPs, conjugated with second antibodies anti-*Salmonella*, were used as labels. At the end of all immunological steps, the modified MBs were captured on the working electrode of a carbon-based SPE, which incorporates a permanent magnet underneath; the electro-reduction of the gold was measured using DPV. This immunosensor detected *S*. Typhimurium in 1.5 h in the concentration range 10^3^–10^6^ CFU/mL with a LOD of 143 CFU/mL. Milk samples were then spiked with two concentrations of *S*. Typhimurium (1.5 × 10^3^ and 1.5 × 10^5^ CFU/mL) to evaluate the recovery percentage. Although good recovery values were calculated (83–94%), we would like to point out that for microbiological criteria, according to EC Regulation 2073/2005, the quantification of *Salmonella* in food is not required. The authors should have demonstrated the effectiveness of the method to take over one viable *Salmonella* cell in 25 mL after an appropriate pre-enrichment time, during which the numerous non target microorganisms, naturally present in the sample, compete with *Salmonella* growth.

**Figure 1:**
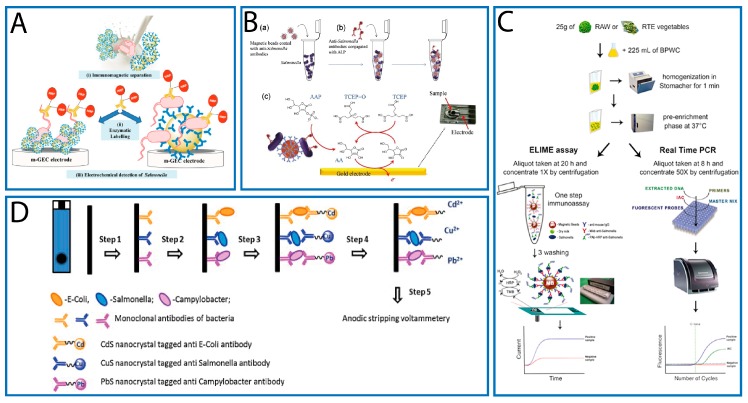
(**A**) *Salmonella* detection based on electrochemical magneto-immunosensing, including: (i) immunomagnetic separation; (ii) enzymatic labeling; and (iii) electrochemical readout (with permission of [45]. (**B**) Immunomagnetic pre-concentration and electrochemical detection based on redox cycling. The detection procedure contained three main steps: (a) immunomagnetic separation and pre-concentration of *Salmonella* from sample matrix; (b) immunological reaction with anti-*Salmonella* antibodies conjugated with alkaline phosphatase; and (c) enzyme reaction and electrochemical detection (with permission of [46]). (**C**) *Salmonella* detection with an ELIME (Enzyme-Linked-Immuno-Magnetic-Electrochemical)-based sandwich assay that involves three sequential procedures: washing-blocking-coating, two sequential incubations for the immuno-recognition events, and the electrochemical detection using eight-well/SPE strips (with permission of [47]). (**D**) Multi-detection of pathogens using NC antibody conjugates and MWCNT-PAH/SPE: Step 1, immobilization of antibodies; Step 2, immunocapture; Step 3, NC-antibody conjugates immunobinding; Step 4, dissolution of metal ions from NC; and Step 5, SWSV analysis (with permission of [48]).

The fabrication of a magneto-electrochemical immunosensor for *S.* Typhimurium was described by Brandao et al. [45]. A comparative study between micro- (2.8 µm) and nano-sized (300 nm) magnetic beads (MMBs and NMBs), coated with monoclonal antibodies against *S*. Typhimurium, was performed (Figure 1A). In both cases, once the immuno-recognition event occurred, a second polyclonal antibody anti-*Salmonella*, labeled with HRP, was added and used as electrochemical reporter, instead of GNPs adopted by Afonso [44]. LODs of 835 and 462 CFU/mL were calculated (in an assay time of 1 h) using micro- and nano-sized MBs, respectively. When a calibration curve for *Salmonella* was constructed in a milk sample, lower LODs (538 CFU/mL for MMBs and 291 CFU/mL for NMBs) were obtained. Although a slightly better LOD was obtained when NMBs were used, the nano-sized MBs displayed an increased matrix effect together with a required longer time for magnetic actuation. Even if the larger surface-to-volume ratio of the NMBs provided more reactive sites for the attachment of higher amounts of bacterial cells, the attachment of 1 µm-bacterial cells was similar for both the nano- and micro-MBs. However, the non-specific adsorptions in complex media appeared higher for the NMBs with respect to the MMBs, NMBs were used to analyze milk samples artificially contaminated with *S*. Typhimurium. These samples were pre-enriched in BHI broth for different times (0, 4, 6, 8 and 10 h) prior to the analysis. The results of this study showed that the immunosensor was able to detect one *Salmonella* cell in 25 mL, after 8 h of pre-enrichment. However, even if a slightly lower LOD was obtained with NMBs, these showed an increased matrix effect in milk. Authors claimed that no conclusive results about the influence of the size of the MBs are present in literature.

An interesting approach based on a two-step strategy, which included immunomagnetic pre-concentration and redox cycling, to amplify the electrochemical signal, was adopted by Wang et al. [46]. In particular, MBs modified with anti-*Salmonella* antibodies were used for separation and pre-concentration of the target bacterium. Then, anti-*Salmonella* antibodies, conjugated with alkaline phosphatase (ALP), were employed to form the sandwich complex. Once the immunological steps are completed, a mixture of ascorbic acid 2-phosphate (AAP) and tri(2-carboxyethyl) phosphine (TCEP) was added to the MBs. ALP catalyzed the conversion of AAP to the electroactive ascorbic acid (AA) and, after 30 min of enzymatic reaction, the solution was transferred onto a gold SPE. At a selected fixed potential, the oxidation of AA occurs. The oxidized AA was then reduced back by the reductant TCEP, allowing additional signal generation at the electrode surface (Figure 1B). The detection limit of this immunosensor, with an analysis time of 1.5 h, was approximately 7.6 × 10^2^ and 6.0 × 10^2^ CFU/mL in broth cultures and agricultural water (which represent the main source of pathogenic contamination of vegetables, including *Salmonella*), respectively. When agricultural water was subject to a pre-enrichment phase of 4 h, the immunosensor was capable to detect a very low *Salmonella* concentration (10 CFU/mL).

Volpe and co-authors [49] also focused their attention on the detection of *Salmonella* in irrigation water. They set-up an ELIME (Enzyme-Linked-Immuno-Magnetic-Electrochemical) assay which involves the formation of a sandwich immunological complex, supported by MBs, and a strip of eight magnetized SPEs (localized at the bottom of eight wells), connected to a portable instrument, allows eight simultaneous amperometric measurements. To have a simple and rapid assay, the authors decided to perform the coating and the blocking steps in a preliminary phase (to store MBs at 4 °C until use), and to merge the two incubations for the immuno-recognition events (the first between monoclonal antibody and *Salmonella*, and the second one between *Salmonella* and a polyclonal antibody conjugated with HRP) in a single step of 1 h. The so-assembled system was able to detect 10^3^ and 2 × 10^3^ CFU/mL of *S.* Napoli and *S.* Thompson (strains responsible for various community alerts), respectively. To demonstrate the reliability of the developed assay, a Real-Time PCR (RTi-PCR) method was also carried out. Unlike other electrochemical biosensors cited in this review, the selectivity of the ELIME assay was largely proved by inclusivity and exclusivity tests performed analyzing different *Salmonella* serotypes and non-target microorganisms. Furthermore, ELIME assay was applied to experimentally and not experimentally contaminated irrigation water samples. Results, compared with those obtained in RTi-PCR and confirmed by the ISO culture method, demonstrated the effectiveness of ELIME to detect a low number of *Salmonella* cells (1–10 CFU/L) after 10 h of pre-enrichment.

Recently, the same authors have demonstrated the applicability of the ELIME assay in fresh (raw and ready-to-eat) leafy green vegetables [47]. For this purpose, 13 not *Salmonella*-contaminated leafy green vegetables (eight raw and five ready-to-eat) were experimentally inoculated with the target bacterium (1–10 CFU/25 g), pre-enriched, and analyzed in parallel with ELIME and RTi-PCR assays. During experimental activity, great attention was paid to the evaluation of the sample matrix effect and to the selection of the medium broth (to be used during the pre-enrichment phase) able to promote a more effective *Salmonella* growth. A confirmation of the ability of both methods to detect such a low *Salmonella* concentration, after 20 h (ELIME) and 8 h (RTi-PCR) of pre-enrichment, was performed with the ISO method. A schematic representation of the procedure adopted, from sample treatment to ELIME and RTi-PCR analysis, is shown in Figure 1C.

An immunoassay format was used to electrochemically detect *S*. Typhimurium [50], according with the biobarcode method developed by Mirkin and co-workers [51].

The concept of the biobarcode is that nano- and micro-sized particles are functionalized with unspecific oligonucleotide strands allowing the particles to be “read”. Firstly, latex spheres were modified with ferromagnetic Fe_3_O_4_ particles. The biobarcode was formed by modifying each sphere with monoclonal antibody against *S.* Typhimurium and single stranded-DNA sequences. *Salmonella* cells were detected by adding the biobarcodes into well plates containing the target bacterium and a biotin-conjugated polyclonal antibody anti-*Salmonella*. After formation of a sandwich-type structure, the biobarcodes were washed and collected on an avidin-modified SPE, allowing them to be covalently bound to the SPE surface by exploiting the interaction between the electrode-confined avidin and the biotin-tagged polyclonal antibody. The excess of biobarcodes (without *Salmonella* and then without the biotinylated sandwich complex) was washed away. Finally, an Ag enhancer solution was loaded onto the SPE and the amount of biobarcodes remaining on the electrode surface (proportional to the antigen concentration) was quantified by Differential Pulse Anodic Stripping Voltammetry (DPASV) measurement of Ag^+^ in acidic solution. The assay, with an analysis time of about 1 h, displayed linearity up to 10^6^ CFU/mL with a very low detection limit (12 CFU/mL). The authors interrogated the assay towards real samples. Green bean sprouts, raw eggs, and plain milk, after appropriate treatments, were spiked with heat-killed and whole cells of *S.* Typhimurium obtaining a LOD of 13 and 26 CFU/mL, respectively. Although encouraging results were attained, the presence of chloride ions represents a serious issue due to the AgCl formation during the Ag enhancement process.

#### 3.1.3. QDs

Viswanathan [48] and Freitas [52] fabricated electrochemical immunosensors exploiting quantum dots as label strategy, although different materials to support the immunological chain were used.

In particular, a disposable electrochemical immunosensor for multiplexed detection of food-borne pathogens (*E*. *coli* O157:H7, *Campylobacter* and *Salmonella*) was proposed by Viswanathan et al. [48]. The immunosensor was fabricated by immobilizing a mixture of antibodies, against the three target bacteria, onto the surface of a multiwalled carbon nanotube-polyallylamine modified SPE (MWCNT-PAH/SPE). Once the antigen–antibody interaction occurred, the sandwich was completed by adding three specific antibodies conjugated with different quantum dots (CdS, PbS, CuS for *E. coli* O157:H7, *Campylobacter* and *Salmonella*, respectively). After a dissolution step, the metallic component of the QDs were released and three well-distinguished current peaks were obtained using Square Wave Anodic Stripping Voltammetry (SWASV), a very effective and widely adopted technique for high sensitivity metal analysis (Figure 1D). The authors demonstrated that MWCNT-PAH/SPE film enhanced the peak currents (if compared to bare SPE and PAH/SPE) due to its particular electrical properties. The analysis time was about 4 h and the detection limit of the assay was found to be 400 CFU/mL for *Salmonella* and *Campylobacter*, and 800 CFU/mL for *E. coli*. The potential of this immunosensor for multiplexed analysis in food samples was proved by the authors analyzing fresh bovine milk spiked with high concentrations (10^4^ CFU/mL) of the three target bacteria.

Freitas et al. [52] proposed a sandwich immunosensor based on the use of SPE, iron oxide/gold core/shell nanomagnetic particles (Fe@GNPs), coated with anti-*Salmonella* antibodies, and CdS nanocrystals, conjugated with the same antibodies, as electrochemical labels. Although this immunosensor was able to detect 13 CFU/mL of *S.* Typhimurium (in less than 1 h), its fabrication requires complex synthesis and functionalization of both Fe@GNPs and CdS nanocrystals, followed by their conjugation with specific antibodies. In addition, in this case, the system was employed to analyze milk samples spiked with 10^3^ and 10^4^ CFU/mL of *Salmonella*, obtaining acceptable recovery.

### 3.2. Label Free Electrochemical Immunosensors

**Figure 2:**
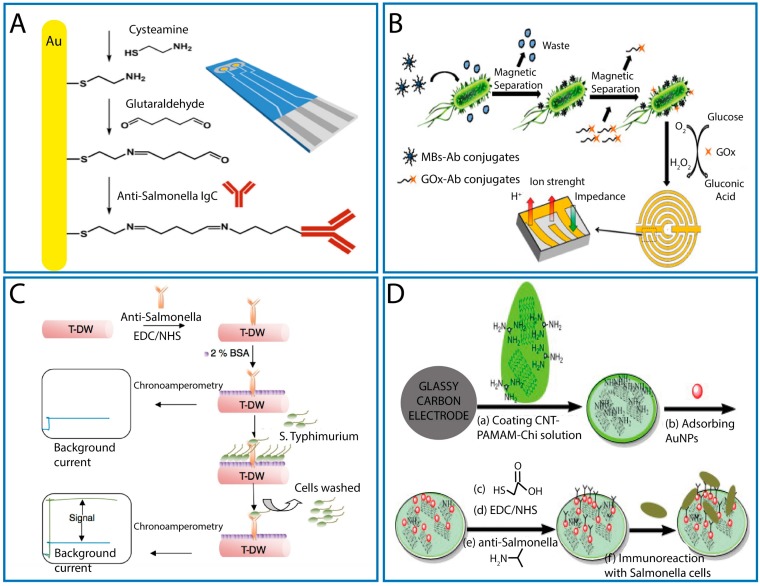
(**A**) Scheme of antibody immobilization and design of the SPE for rapid immunosensing of *S.* Typhimurium using electrochemical impedance spectroscopy (with permission of [53]); (**B**) mechanism and construction of the impedimetric immunosensor based on the use of magnetic beads for separation and rapid detection of *E. coli* O157:H7 and *S.* Typhimurium in foods (with permission of [54]); (**C**) scheme of the electrode platform based on label-free as-grown double wall carbon nanotubes bundles for *S.* Typhimurium immunoassay (with permission of [55]); and (**D**) schematic processes of the immunosensor fabrication process: (a) dropping of PAMAM-MWCNT-Chit membrane; (b) assembly of AuNPs; (c) assembly of mercaptoacetic acid; (d) activation of carboxyl groups with EDC/NHS; (e) capture of anti-*Salmonella* antibodies; and (f) immunoreaction of anti-*Salmonella* and *Salmonella* cells (with permission of [56]).

A gold two-channel SPE was used, by Skladal’s group [53], to fabricate a label-free electrochemical immunosensor without the employment of nano- and micro-sized materials. In particular, the specific antibody (against the lipopolysaccharides-LPS of *Salmonella*) was immobilized on one of the two gold electrodes via cysteamine monolayer activated with glutaraldehyde (Figure 2A), followed by a blocking step with albumin serum bovine (Au-cys-GA-Ab/BSA). The second gold electrode, Au-cys-GA-BSA, was used as blank. After the binding event, the impedance spectra were measured between the two gold electrodes, using [Fe(CN)_6_]^3−/4−^ as redox probe. When the immunosensor, with a total analysis time of 20 min, was tested on heat-treated and sonicated *Salmonella* cells, a linear range up to 10^8^ CFU/mL of *S*. Typhimurium and a detection limit down to 10^3^ CFU/mL were obtained. Only pure broth cultures of *E. coli* were analyzed to evaluate the specificity of the immunosensor and a negligible interference was observed. Finally, milk samples were fortified with known amounts of *S*. Typhimurium, and the effect of cell treatment (heating, sonication, and a combination of both), on the sensor response, was re-evaluated. Different LODs were calculated: 7 × 10^4^, 6 × 10^2^ and 10^3^ CFU/mL, respectively. The authors concluded that all the adopted treatments were useful to reveal the infection dose of *Salmonella*, but they understate that it is not important to detect the infection dose in foods, but rather to establish the absence of this pathogen in 25 g or 25 mL of food product.

#### 3.2.1. MBs

An easy to fabricate impedimetric immunosensor was described by Xu et al. [54] for the detection of *S.* Typhimurium. It was based on the use of MBs for separation and a screen-printed interdigitated microelectrode (SP-IDME) as transducer. MBs, coated with streptavidin, were firstly functionalized with specific biotinylated antibodies and then added in a bacterial suspension for the binding event. Once the target bacterium was captured, second specific antibodies, conjugated with glucose oxidase enzyme (Ab-GOx), were used to label the bound bacteria by forming a MBs-Ab-cell-Ab-GOx sandwich complex. The yielded MBs-Ab-cell-Ab-GOx complex was mixed with a glucose solution to trigger an enzymatic reaction which produced gluconic acid. This increased the ion strength of the solution, thus reflecting in a decrease of the impedance measured at SP-IDME (Figure 2B). The impedimetric immunosensor was able to measure (in about 2 h) 10^2^–10^6^ CFU/mL of *S.* Typhimurium, with a LOD of 1.66 × 10^3^ CFU/mL. In addition, in this paper, the specificity of the assay was poorly demonstrated by testing a low number of non-target bacteria. The detection of *S.* Typhimurium in chicken carcass rinse water was examined. For this purpose, samples (after an ad hoc treatment) were inoculated with controlled concentrations of the target bacterium verifying the capability of the method to detect 10^3^ CFU/mL of *S.* Typhimurium. The easy-to-operate system was extended to another pathogenic bacterium (*E. coli*), replacing only the specific antibodies.

#### 3.2.2. CNTs

Punbusayakul [55] and Liu [57] developed label-free electrochemical immunosensors with amperometric detection, exploiting CNTs to modify the electrode surface.

Punbusayakul and colleagues reported the first use of as-grown double walled carbon nanotubes bundles for fabricating a label-free immunosensor for *S.* Typhimurium detection [55]. This immunosensor was constructed by using as-grown, horizontally aligned, thread-like and double-walled (T-DW) CNTs. An anti-*Salmonella* monoclonal antibody was covalently immobilized onto the T-DW modified electrode by using the EDC crosslink method, and BSA as blocking agent. The so fabricated immunosensor was stable if stored at 4 °C in humid condition. Once the binding event occurs (2 h), a chronoamperometric measurement was carried out exploiting the typical negative charged cell wall of the gram-negative bacteria, which contains lipopolysaccharide (Figure 2C). A linear range between 10^2^ and 10^7^ CFU/mL and a LOD of 8.9 CFU/mL were obtained. The immunosensor was only tested for non-specific sensing vs. *E. coli* and was not interrogated in real samples.

A novel self-made portable amperometric biosensor, based on SPE modified with multi-walled carbon nanotubes-chitosan-peroxidase, for the detection of *S.* Pullorum in chicken samples was proposed by Liu et al. [57]. In this study, anti-*Salmonella* polyclonal antibodies were immobilized on cellulose nitrate membranes, placed on the bottom of 24 wells of a plastic tissue culture plate. During use, antigen–antibody interaction took place in the wells, and then H_2_O_2_ and TMB were added. The activity of the catalase enzyme, a biomarker of *S.* Pullorum, which catalyzes the H_2_O_2_ dismutation, was amperometrically monitored by adding the reaction product on the SPE modified with multi-walled CNT-chitosan-peroxidase. This method allowed detecting 100 CFU/mL of *S.* Pullorum within 30 min. When three *S.* Pullorum-free chicken samples were inoculated with the target bacterium and pre-enriched in a nutrient broth for different times, the amperometric sensor was able to detect 60–100 CFU/mL after 1.5–2 h of pre-enrichment. This result suggests that the method has all the potentials to detect a viable *Salmonella* cell after a short pre-enrichment time. Although the authors stated that catalase is a biomarker of *S*. Pullorum, we want to underline that this enzyme is present not only in the genus *Salmonella* but also in other bacteria such as *Bacillus cereus*, *Staphylococcus aureus*, *E. coli* (very frequently found in meat products), *Pseudomonas aeruginosa*. For this reason, the authors should have to carry out inclusivity and exclusivity tests to demonstrate the ability of their method to detect different *Salmonella* serotypes avoiding false positive results, due to the possible interaction between the polyclonal antibodies and other non-target bacteria.

Dong et al. [56] developed a label free electrochemical impedance immunosensor for the detection of *S*. Typhimurium using a poly(amidoamine)-multiwalled carbon nanotubes-chitosan nanocomposite film modified glassy carbon electrode (GNPs/PAMAM-MWCNT-Chit/GCE). In addition, GNPs were employed as support to immobilize anti-*Salmonella* antibodies. In particular, the surface of a bare GCE was coated with a PAMAM dendrimer-MWCNTs-Chit solution, and then GNPs were attached to PAMAM dendrimer via amido-gold affinity. Thanks to the large surface of PAMAM dendrimer it was possible to load a large amount of GNPs and, consequently, an increased number of anti-*Salmonella* antibodies were immobilized (via EDC/NHS) onto the gold nanoparticles (Figure 2D). After the binding event, between antibodies and *Salmonella* cells, the electron transfer resistance (R_ct_) was directly measured by EIS, using [Fe(CN)_6_]^3−/4−^ as a redox probe. Although the biosensor fabrication is quite complex and requires a long preparation time, a sensitive (LOD of 5 × 10^2^ CFU/mL) and stable (four weeks at 4 °C) label free immunosensor was assembled thanks to the combination of these innovative materials (GNPs/PAMAM-MWCNT-Chi). The specificity of the immunosensor was only proved by testing *E. coli* and *S. aureus*, as interfering bacteria, at 10^6^ CFU/mL. Finally, the immunosensors, with an analysis time of 1 h, was applied in three fat-free milk samples experimentally contaminated with 10^3^, 10^5^, 10^7^ CFU/mL of *S.* Typhimurium obtaining satisfactory recoveries from 94.5% to 106.6%.

#### 3.2.3. GR

Mutreja et al. [58] reported, for the first time, the use of a novel specific outer membrane antigen (OmpD) to develop a selective and sensitive impedimetric immunosensor to detect *S.* Typhimurium in water and juice samples. Anti-OmpD antibodies, against this specific surface antigen, were produced and attached to the reduced graphene–graphene oxide (rG-GO), modified SPE, by EDC/NHS chemistry. EIS was employed to monitor impedance changes, in 5 mM potassium ferrocyanide, due to the specific binding between antibodies and *Salmonella* cells (10–10^6^ CFU/mL), obtaining a detection limit of ~10 CFU/mL. A similar LOD was calculated analyzing water and juice samples (lichi and orange juices) fortified with known amount of the target bacterium. Based on this result, the authors claimed that no pre-enrichment step is required before sample analysis. We want to underline that this LOD (250 CFU/25 mL) is higher than 1 CFU/25 mL (minimum concentration to be detected in fruit juices to establish the positivity of the sample); therefore, the pre-enrichment phase is necessary both to increase the number of *Salmonella* cells (until reaching the detection limit of the method) and to distinguish between living and dead cells.

The main features of label-based and label free electrochemical immunosensors for *Salmonella* detection are reported in Table 1.

## 4. Electrochemical Genosensors, Phagosensors and Aptasensors for *Salmonella* Detection

This section is devoted to illustrate the remaining electrochemical platforms that are mainly used for the detection of bacteria, namely genosensors, phagosensors, and aptasensors.

Specifically, genosensors represent wide and effective tools for the construction and assembling of sensitive platforms. They are based on the use of genetic building blocks as probe, while the target is typically represented by a specific gene, extracted from the bacterial cells. The main advantage of this biosensor class, if compared with the immunosensors (based on the use of antibodies), relies on the synthesis and storage easiness and the high stability of the biorecognition probes. The classical genosensor is based on the measurement of the hybridization event between the target single stranded-DNA (ssDNA) and the complementary capture probe, attached to the electrode surface or, alternatively, linked to external supports (i.e., MBs). However, this approach requires DNA amplification techniques, such as PCR, to amplify only the target gene of the pathogen: the so-formed amplicon, after being denatured and cooled, hybridizes (as ssDNA) with the capture probe to form double stranded-DNA (dsDNA). In this regard, we want to specify that some authors, working in the genosensor field, do not report the amplification phase because they use only synthetic ssDNA [59,60,61]. The analysis of the target bacterium, instead of synthetic ssDNA, therefore requires a preliminary cell lysis (for the release of the genomic DNA), followed by amplification.

Besides the genosensors, phagosensors represent another option in the development of electrochemical platforms. A bacteriophage, or phage, is a virus that is capable to specifically infect a target bacterium. For analytical biosensing development, three main categories are employed: (i) lytic phages that specifically release the content of a bacterial strain; (ii) non-lytic phages that are functionalized to immobilize the target; and (iii) genetically engineered lytic and non-lytic phages that allow the bacteria to produce target metabolites, i.e., uncharged substrates that can be metabolized into acids, producing a change of the impedance/conductivity of the working solution [62,63,64]. To the best of our knowledge, only an example of phagosensor has been developed to electrochemically detect *Salmonella* over the last five years [65]. In this case, described in detail within the dedicated session of this review, the bacteriophage behaves as biorecognition element for the target cells causing their lyses, with consequent release of the genomic DNA. As well as for the genosensors, the target gene, after amplification, denaturation and cooling, hybridized with a complementary ssDNA capture probe, immobilized on the electrode.

Aptasensors, short oligonucleotides selected through an entirely in vitro combinatorial biochemistry method (SELEX), are capable to directly detect whole cells, allowing one-step and PCR-free approaches. Aptamers selectively recognize and bind whole cells through a conformational change of their structure. If compared to their “natural” counterpart (antibodies), aptamers can be synthesized with high reproducibility and purity, and appear more stable. Aptamers-based electrochemical biosensors for *Salmonella* detection are mainly coupled with electrochemical impedance spectroscopy, allowing a label free approach.

In this review, as for electrochemical immunosensors, genosensors, phagosensors, and aptasensors have been divided in two main subclasses: label-based and label free. The former requires a marker (that may itself be electroactive or able to generate an electroactive product) attached to the capture probe or to the target in order to achieve an electron-transfer. The latter normally requires the addition of an external redox probe (detected by EIS or voltammetric techniques) or the application of small DNA-intercalating electroactive compound that gives the possibility to distinguish a single-stranded DNA probe from a double-stranded hybrid DNA, located at the electrode surface. Moreover, conductive polymers, which represent very attractive materials for the fabrication of functional interfaces and sensing surface, can be used to detect the binding event through the intrinsic variation of their electrical properties, without addition of a redox probe [66].

### 4.1. Label-Based Approaches

#### 4.1.1. Genosensors

Li et al. [67] detected *Salmonella* by extracting genomic DNA. The *invA* gene (highly conserved in almost all *Salmonella* serotypes), after PCR amplification and thermal denaturation, was specifically detected at the surface of a gold electrode, previously modified with a ssDNA capture probe. Once the hybridization event occurred, a detection probe, functionalized with alkaline phosphatase, was added to form a sandwich-type structure. *Salmonella* was indirectly detected by exploiting the hydrolysis of α-naphthyl phosphate (enzymatic substrate), yielding α-naphthol that was measured by DPV. The mismatched oligonucleotides were satisfactorily discriminated, suggesting a good selectivity of the developed method. Without the use of a pre-enrichment step, *S.* Typhimurium was detected in the range between 10 and 10^5^ CFU/mL. Although the detection limit of 10 CFU/mL and an analysis time of 3.5 h represent satisfactory features, the proposed method did not take into account the analysis of real samples.

##### GNPs

**Figure 3:**
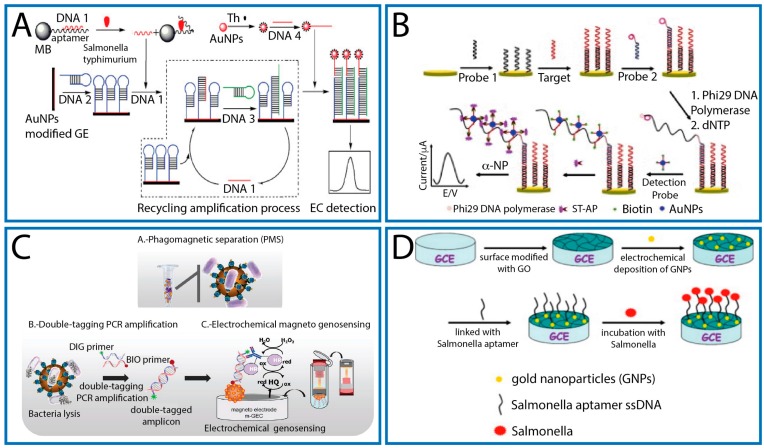
(**A**) Schematic illustration of signal amplified strategy based on entropy-driven molecule switch and EC nanoparticle probe for DNA and *Salmonella* Typhimurium detection (with permission of [68]); (**B**) the electrochemical sensing strategy developed for rapid detection of *Salmonella* by combining the rolling circle amplification with DNA–AuNPs probe (with permission of [69]); (**C**) schematic representation of the phagomagnetic separation of the bacteria followed by the double-tagging PCR, and the electrochemical magneto-genosensing of the attached bacteria (with permission of [65]); and (**D**) schematic illustration for the aptamer-based electrochemical detection of *Salmonella* based on a GO/GNPs modified glassy carbon electrode (with permission of [70]).

Zong et al. developed a new methodology based on entropy-driven molecule switch signal amplification strategy to detect *S.* Typhimurium [68]. GNPs were employed both to enhance the electrochemical performance of a gold electrode and also as labels. In particular, the GNPs (deposited onto the electrode surface) were used to immobilize a capture hairpin DNA. In the presence of a synthetic complementary DNA target of *S.* Typhimurium, the stem of the capture hairpin DNA was opened as consequence of the hybridization event. A link DNA, which had more complementary bases with the capture DNA, was added to hybridize with the capture DNA, causing the displacement of the target (under an entropy-driven mechanism). The released DNA was then able to open another capture probe immobilized onto the electrode surface. Thanks to a series of recycling events (4 h), a low target concentration was able to open a large amount of capture probe. Finally, the addition of a detection probe (containing thionine/GNPs composite) capable to bind a complementary sequence of the hybridized link DNA, allowed the electrochemical detection by DPV (Figure 3A). The genosensor displayed a very low detection limit equal to 0.3 fM for DNA of *S.* Typhimurium.

Zhu et al. [69] combined, for the first time, the Rolling Circle Amplification (RCA) and DNA-GNPs. Different from other works [68,71], GNPs were not employed to modify the gold electrode because of its already satisfying conductive properties. A thiolated capture probe was immobilized onto the surface of a bare gold electrode and allowed to hybridize with a suitably ssDNA target (obtained amplifying *Salmonella invA* gene by PCR). Then, a circulation mixture, containing a circular DNA template was added to form a typical sandwich structure. Thanks to the presence, in the circulation mixture, of other reagents (i.e., DNA polymerase), the RCA occurs generating a long ssDNA molecule with repetitive sequence units that are complementary to the circular DNA template. These sequences act as repeated anchor points for the attachment of a large amount of the recognition probes, i.e., DNA-GNPs conjugated with biotin; the subsequent addition of streptavidin-ALP/α-naphthyl phosphate allowed to obtain a DPV signal-on (Figure 3B). The presence of GNPs produced a three-fold increase of the method sensitivity. The calibration curve of the target DNA sequence displayed good linearity from 10 aM to 10 pM with an ultra-low detection limit of 6.76 aM. Following these findings, *Salmonella*-spiked milk samples were analyzed (after removing lipids and proteins) and a LOD of 6 CFU/mL was calculated. The specificity was evaluated in presence of three interfering PCR products of *Escherichia coli*, *Staphylococcus aureus*, and *Streptococcus pneumonia*, and, in each case, the biosensor displayed excellent selectivity for *Salmonella* detection. 

##### MBs

In 2016, the research group of Pividori utilized, for the first time, silica magnetic particles as carrier in electrochemical magneto-genosensing of single-tagged amplicons (produced by PCR after the release of *Salmonella* DNA) [72]. Two specific primers were employed for the amplification of the *invA* gene, being one of the two primers tagged with fluorescein. After PCR amplification, the single tagged-amplicons were immobilized on silica magnetic particles and they were enzymatically labeled using antibodies, anti-fluorescein conjugated with HRP. Several washing steps were necessary to lower the interferences and then the silica particles were concentrated on the magneto electrode. This genosensor was capable to amperometrically detect down to 0.04 ng/µL of the target sequence (the number of *Salmonella* cells was not specified). Three hours, including PCR amplification, were enough to complete the assay. The use of other specific primers allowed detecting the target genes of different pathogenic bacteria, i.e., *L. monocytogenes, E. coli.* Even if the sensing approach was similar to that developed previously from the same group [65], the authors stressed the fact that the use of silica magnetic particles allowed to detect selectively longer dsDNA fragments reducing the interferences due to the shorter ssDNA primers. This depends on the fact that DNA is weakly attracted to silica by non-specific forces, but ds amplicons resulted better anchored than the ss primers, thanks to the higher negative charge of the sugar–phosphate backbone. However, no real samples were interrogated.

#### 4.1.2. Phagosensors

##### MBs

Liébana et al. coupled the effectiveness of bacteriophages with electrochemical magneto-genosensing [65]. *Salmonella* (serogroups A, B, and D1) was specifically recognized and separated with P22 bacteriophage-modified MBs. Once the *Salmonella* cells were captured on the MBs, they were thermally lysed to release the genomic DNA. Double-tagging PCR amplification was carried out using two specific modified primers (3′Dig-primer and 5′biotin-primer) and the resulting double-tagged amplicons were captured by streptavidin magnetic beads. Antibodies, anti-Dig conjugated with HRP, were used to bind the 3′Dig ends of the ds-DNA amplicons. The modified magnetic beads were finally captured on the surface of a magneto electrode for the amperometric detection (Figure 3C). This strategy was capable to detect in 4 h as low as 3 CFU/mL of *Salmonella*, with a linearity extended up to 10^5^ CFU/mL. This platform compared favorably, in term of LOD, with other approaches that used bacteriophages [73,74]. As proposed by the authors, the combination between magnetic separation and amplicon detection played a crucial role in lowering the LOD. Although this platform was not interrogated for food samples, but only in Luria-Bertani broth, the addition of a pre-enrichment step could be consistent with the detection of one viable *Salmonella* cell in an established amount of food product.

#### 4.1.3. Aptasensors

##### GNPs

In 2016, after having developed a genosensor for *Salmonella* [67], Li and co-workers proposed an electrochemical aptasensor based on target-induced strand displacement approach [71]. They took advantage of GNPs both as electrode modifier and assembled with the detection probe. The use of GNPs as electrochemical enhancer has led to a tiny increase of the sensitivity (two-fold) with respect to the bare gold electrode. In the presence of the target bacterium, the aptamer was displaced from the capture probe by binding the pathogen and, successively, the addition of a detection probe/gold nanoparticles/alkaline phosphatase structure allowed the capture probe to be hybridized. As for their previous work [50], the electrochemical signal was recorded following the oxidation of the alkaline phosphatase by-product in presence of α-naphthyl phosphate (substrate). A linear dynamic range from 20 to 2 × 10^6^ CFU/mL and a detection limit of 20 CFU/mL were obtained. When the aptasensor was employed to analyze spiked milk samples, a LOD equal to 200 CFU/mL was calculated.

##### MBs

Zong et al., starting from the convincing results obtained by involving the entropy-driven molecule switch strategy, utilized the same displacement reactions to detect *S*. Typhimurium whole cells. In this case, magnetic beads were used to immobilize *S*. Typhimurium aptamer and then a complementary sequence was hybridized with the aptamer [68]. When *S*. Typhimurium was added, the whole cell bound its aptamer and the complementary sequence was released. The solution containing the released DNA was detected by the signal amplification method already described for the genosensor, and a detection limit of 13 CFU/mL was reached. Milk samples (centrifuged and diluted 20-fold with distilled water) were spiked with known concentration of the target bacterium (56 to 1110 CFU/mL) and analyzed, obtaining good recoveries.

### 4.2. Label-Free Approaches

#### 4.2.1. Genosensors

Garcia and colleagues developed an electrochemical device, exploiting the Micro Electro-Mechanical Systems (MEMS) technology to fabricate thin-film gold electrodes [59]. Even if, as claimed by the authors, photolithography allowed obtaining a surface two-fold rougher than the commercial ones (DropSens S.L., Llanera, Spain), the three required photolithographic and sputtering processes make this approach quite expensive. A synthetic oligonucleotide sequence of *Salmonella* was selectively detected after its hybridization (1 h) with a ssDNA, previously immobilized on the gold surface. The use of an intercalating ruthenium-based redox probe allowed the quantification of the target sequence (by DPV) from 5 to 30 µM with a LOD of 0.208 µM. In addition, in the presence of interfering synthetic sequences from other pathogens, i.e., *Lysteria* and *Escherichia coli*, this platform was proved to be selective for the target and this feature drove the authors to develop a simultaneous array sensor to detect *Salmonella*, *Lysteria* and *E. coli* in the same solution, using different probes. Even if the selectivity of the different probes resulted promising, this device was not challenged neither in bacteria culture broths (pure or mixed) nor in food samples.

Tabrizi and colleagues proposed a dual-technique genosensor for the detection of *Salmonella* [60]. A synthetic oligonucleotide sequence of *Salmonella* was used as target to be captured by a complementary immobilized DNA. In order to both increase the roughness and generate carboxylic acid groups on a glassy carbon electrode surface, an electrochemical oxidation/reduction cycle was carried out. It allowed attaching a higher amount of single stranded DNA, as capture probe. After an hybridization time of 45 min, DPV and EIS approaches, using Fe(CN)_6_^3−^ as redox probe, were carried out. DPV provided a response linear from 10 to 400 pM with a detection limit of 2.1 pM, while the impedimetric measurement gave a wider linear range, from 1 to 400 pM, and a lower detection limit, equal to 0.15 pM. These results are consistent with the principles of the electrochemical techniques that have been used: EIS is a very sensitive technique and, with respect to DPV, it displays a signal-on response, while DPV is characterized by a decrease of the signal, representing a signal-off approach. The platform was not tested, neither in *Salmonella* broth cultures nor in real samples.

##### GNPs

Das and co-workers utilized a disposable carbon-based SPE to develop a sensitive platform for *S.* Typhimurium detection, employing *Vi* gene as molecular marker [61]. GNPs were electrochemically deposited onto the SPE and then, a single stranded DNA probe was attached to their large surface. After 60 min of hybridization with the target DNA, methylene blue intercalated in the formed dsDNA structure, producing an increase of the DPV signal that was proportional to the target level. The strategy that was adopted in this work allowed to assemble a signal-on device, which was consistent with a LOD of 50 × 10^−12^ M and a linearity up to 0.5 × 10^−8^ M. The cost-effective feature of the screen-printed platform was further highlighted by its suitability in being reusable up to four times with about a 15–20% loss of the original signal. Although this biosensor was successfully applied in 10%-diluted serum samples, spiked with different concentrations of the synthetic single stranded DNA, nether *Salmonella* broth cultures nor food samples were taken into account.

#### 4.2.2. Aptasensors

##### GNPs

Ma and colleagues took advantage of graphene oxide (GO) and GNPs to modify a glassy carbon electrode [70]. While GO was drop cast on the working electrode, GNPs were formed electrochemically. The presence of the hybrid nanoplatform improved the immobilization of the capture probe, a single stranded DNA aptamer specific for *Salmonella* (serotype not indicated), and enhanced the electron transfer at the electrode. The electrochemical impedance spectroscopy (EIS) was adopted to quantify the cells after 90 min of incubation on the modified electrode (Figure 3D). This method avoids the use of time/reagent consuming procedures as for biosensors that require PCR amplification. This platform reached a detection limit as low as 3 CFU/mL and it was linear until 2.4 × 10^3^ CFU/mL. Experiments carried out in presence of different bacteria such as *Listeria monocytogenes*, *Bacillus subtilis*, *Staphylococcus aureus*, *Streptococcus pyogenes*, *Escherichia coli*, *Enterobacter sakazakii*, confirmed the high specificity of the developed aptasensor towards *Salmonella*. Commercial pork meat was then employed to conduct recovery experiments. In this regard, deep muscle samples were treated, diluted and then spiked with 10^2^–10^4^ CFU/mL of *Salmonella*. Recovery values close to 100% were obtained.

EIS was also adopted by the group of Professor Turner which fabricated an aptamer-based biosensor for the detection of *Salmonella* Typhimurium [75]. This work represents the consequence of a proof of concept developed in 2012 in which GNPs-SPE and specific aptamers were used to fabricate an impedimteric sensor able to distinguish between live and killed bacteria [7]. Here, the aptamer was grafted onto a commercial carbon-based SPE by means of diazonium salt instead of the GNPs. More specifically diazonium salt was grafted by following two strategies: electrochemically and chemically Zn-mediated. The former grafting procedure allowed to fabricate a surface with a higher density of aptamers (10-fold higher than the Zn-mediated procedure), reflecting a higher sensitivity. After a 30-min incubation with the target bacterium and a 15-min washing, the aptasensor was capable to detect *Salmonella* down to 6 CFU/mL, being linear in the 10–10^8^ range (using the redox probes, ferri/ferrocyanide ions). The aptasensor was then applied in undiluted apple juice samples spiked with *S.* Typhimurium concentrations ranging from 10^2^ to 10^6^ CFU/mL, obtaining satisfactory recoveries.

##### Conductive Polymers

The same year, Sheikhzadeh et al. [66] developed another impedimetric biosensor obtained from the combination of polypyrrole-based polymer and an anti-*Salmonella* Typhimurium aptamer. Respect to the previous aptasensor based on the diazonium-functionalized carbon-SPE [75], a gold disk was firstly modified with polypyrrole and then with a *Salmonella*-specific aptamer, obtaining a slightly lower detection limit (3 CFU/mL), with 15 min detection time. It should be considered that, besides its expensiveness, the gold disk required mechanic, ultrasonic, and electrochemical cleaning procedures before starting with the biosensor fabrication. However, the advantage in using polypyrrole, that is a conductive polymer, consisted in the assembling of an entirely label-free platform. In addition, in this work, the aptasensor was interrogated towards apple juice samples that were spiked with 10^2^–10^6^ CFU/mL of *S*. Typhimurium.

The main features of label-based and label free electrochemical genosensors, phagosensors, and aptasensors for *Salmonella* detection are reported in Table 2.

## 5. Conclusions

The detection of *Salmonella* according to EC regulation is still primarily based on traditionally microbiological culture methods that take days to be completed. The development of rapid, sensitive and specific methods to detect food-associated *Salmonella* remains challenging for ensuring food safety. The most recent electrochemical biosensors could represent good candidates to meet this challenge. Even if the reviewed papers have displayed some limitations in food analysis, many are the advantages of the developed electrochemical platforms for detecting foodborne pathogens as well as *Salmonella*: (1) printing technologies, i.e., screen-printed electrodes, continue to provide the end-users wider accessible detecting tools; (2) novel and customizable nano/micro materials, i.e., GNPs, CNTs, QDs, and conductive polymers, enhance the analytical performance of the sensing platforms; and (3) electroanalysis allows moving towards the development of methods that can be “laboratory-free”, i.e., impedimetric detection of whole cells, particularly useful in those resource-limited environments.

This review aims to illustrate that these powerful strategies, which have been adopted by some authors to develop rapid and sensitive electrochemical immunosensors, genosensors, aptasensors and phagosensors, may be finely engineered to allow the detection of very low *Salmonella* levels. Despite the high sensitivity of these tools, they do not distinguish between living and dead cells. This aspect is of great importance as, for microbiological criteria (according to EC Regulation 2073/2005), viable *Salmonella* cells must be absent in a defined amount of a given food product. Therefore, in this context, we have underlined the necessity to couple these systems with a more or less short pre-enrichment phase (during which microbial growth occurs), depending on the sensitivity of the method. Bacteriophages may be a valid alternative to avoid the pre-enrichment phase: phages are viruses able to specifically recognize viable bacterial cells. In addition, as demonstrated in a recent paper, specifically-designed aptamers have displayed the capacity to discriminate between viable and heat killed *Salmonella* cells. Nevertheless, to date, the detection limit of phagosensors and aptasensors does not allow detecting one *Salmonella* cells in an established amount of food product. Therefore, the pre-enrichment step is still required to allow the *Salmonella* to grow up to a detectable level.

Another important aspect that we have emphasized is that the most of the biosensors, cited in this review, are focused on the detection of a single *Salmonella* serotype (i.e., *S*. Typhimurium and *S*. Pullorum) and their ability to reveal different *Salmonella* serovars has not be demonstrated. Thus, the selection of a biological recognition element able to interact with the most common *Salmonella* serotypes, isolated from food, is of crucial importance. Certainly, genosensors, which measure the hybridization event between a target ssDNA and the complementary capture probe (immobilized on the electrode surface or linked to an external support), are those that best meet this requirement. However, these biosensors require a preliminary amplification of the target gene (highly conserved in all *Salmonella* serotypes) by PCR.

In the most of the reviewed papers, the authors have demonstrated the selectivity of the developed biosensors by testing only a limited number of non-target bacteria, and their effectiveness to detect *Salmonella* in food samples was proven by recovery tests (although the quantification of this pathogen is not required). Only some research groups have analyzed blank samples (*Salmonella* free) experimentally contaminated with one *Salmonella* cell and have established the minimum pre-enrichment time necessary to reveal it. For a more extended application of the biosensors in food analysis, is also necessary to analyze not experimentally contaminated samples, and the results obtained (in terms of positive and negative samples) have to be confirmed by the official cultural methods. Thus, we believe that, only through a fruitful collaboration between research groups working in biosensor and food microbiology fields, these powerful analytical tools could be used as screening methods to help the implementation of the HACCP planes in food production industry, improving the level of public health and reducing the economic losses related to the withdrawal of contaminated foods.

## Figures and Tables

**Table 1:** Main features of label-based and label-free electrochemical immunosensors for *Salmonella* detection.

Platform	Nano- and Micro-Sized Materials	Electrochemical Technique	Detection Time	LOD in Broth Cultures CFU/mL	Food Analysis	Reference
GCE	GNPs	DPV	4 h	5	-	[42]
SPE	GNPs	CV	1 h 20 min	3000	Eggs and chicken meat Accuracy 80–100%	[43]
SPE	MBs/GNPs	DPV	1 h 30 min	143	Milk Recovery 83–94% (1.5 × 10^3^–1.5 × 10^5^ CFU/mL)	[44]
GCE	NMBs	Amperometry	1 h	462	Milk LOD 291 CFU/mL LOD 1 CFU/25 mL (8 h pre-enrichment) Milk LOD 538 CFU/mL	[45]
	MMBs			835		
G-SPE	MBs	Amperometry	1 h 30 min	760	Agricultural water LOD 6 × 10^2^ CFU/mL LOD 10 CFU/mL (4 h of pre-enrichment)	[46]
8-SPE strip	MBs	Amperometry	1 h	1000	Irrigation water LOD 1 CFU/L (10 h of pre-enrichment)	[49]
8-SPE strip	MBs	Amperometry	1 h	1000	Fresh leafy green vegetables LOD 1 CFU/25 g (20 h of pre-enrichment)	[47]
SPE	MBs	DPASV	1 h	12	Green bean sprouts, egg, milk LOD 13–26 CFU/mL	[50]
SPE	QDs/CNTs	SWASV	4 h	400	Milk Recovery 95% (10^4^ CFU/mL)	[51]
SPE	MBs/QDs	SWASV	<1 h	13	Milk Recovery 77.6–77.8% (10^3^–10^4^ CFU/mL)	[48]
G-SPE	-	EIS	20 min	<1000	Milk LOD 6 × 10^2^ CFU/mL	[52]
SP-IDME	MBs	EIS	2 h	1660	Chicken carcass rinse water LOD 10^3^ CFU/mL	[53]
T-DW	CNTs	Amperometry	2 h	8.9	-	[54]
SPE	CNTs	Amperometry	30 min	100	Chicken meat LOD 60–100 CFU/mL (1.5–2 h of pre-enrichment)	[57]
GCE	GNPs/CNTs	EIS	1 h	500	Milk Recovery 94.5–106.6% (10^3^–10^7^ CFU/mL)	[56]
SPE	rG-GO	EIS	3 h	10	Water and fruit juices LOD 10 CFU/mL	[58]

List of abbreviations. GCE: glassy carbon electrode; SPE: carbon screen-printed electrode; G-SPE: gold screen-printed electrode SP-IDME: screen-printed interdigitated microelectrode; T-DW: thread-like double walled carbon nanotubes; GNPs: gold nanoparticles; MBs: magnetic beads; NMBs: nano-sized magnetic beads; MMBs: micro-sized magnetic beads; QDs: quantum dots; CNTs: carbon nanotubes; rG-GO: reduced graphene–graphene oxide; DPV: differential pulse voltammetry; CV: cyclic voltammetry; DPASV: differential pulse anodic stripping voltammetry; SWASV: square wave anodic stripping voltammetry; EIS: electrochemical impedance spectroscopy.

**Table 2:** Main features of label-based and label-free electrochemical genosensors, phagosensors, and aptasensors for *Salmonella* detection.

Platform	Nano- and Micro-Sized Materials	Electrochemical Technique	Detection Time	LOD in Broth Cultures	Food Analysis	Reference
GE ^A^	-	DPV	3 h 30 min	10 CFU/mL (after PCR)	-	[67]
GE ^A^	GNPs, thionine/GNPs	DPV	5 h	0.3 fM	-	[68]
GE ^B^	MBs	DPV	4 h 25 min	13 CFU/mL	Milk Recovery 96.1–103.0% (57–1093 CFU/mL)	[68]
GE ^A^	GNPs	DPV	1 h	6 CFU/mL (after PCR)	Milk LOD 6 CFU/mL	[69]
m-GEC ^A^	Silica MBs	Amperometry	3 h	0.04 μg/mL (after PCR)	-	[72]
m-GEC ^C^	MBs	Amperometry	4 h	3 CFU/mL (after PCR)	-	[65]
GE ^B^	GNPs	DPV	3 h 30 min	20 CFU/mL ^D^	Milk LOD 200 CFU/mL	[71]
Thin-film GE ^A^		DPV	1 h	0.208 μM ^D^	-	[59]
GCE ^A^	-	DPV	1 h	2.1 pM ^D^	-	[60]
		EIS		0.15 pM ^D^		
SPE ^A^	GNPs	DPV	1 h 5 min	50 pM ^D^	-	[61]
GCE ^B^	GO/GNPs	EIS	1 h 30 min	3 CFU/mL	Pork meat Recovery 97.3–105% (10–1000 CFU/mL)	[70]
SPE ^B^	-	EIS	45 min	6 CFU/mL	Apple juice Recovery 300–440% (10^2^–10^6^ CFU/mL)	[75]
GE ^B^	PPy	EIS	1 h	3 CFU/mL	Apple juice Recovery 140–410% (10^2^–10^6^ CFU/mL)	[66]

List of symbols and abbreviations. ^A^: genosensor; ^B^: aptasensor; ^C^: phagosensor; ^D^: synthetic DNA; GE: gold electrode; m-GEC: magneto graphite–epoxy composite; GCE: glassy carbon electrode; SPE: carbon screen-printed electrode; GNPs: gold nanoparticles; MBs: magnetic beads; GO: graphene oxide; PPy; polypyrrole; DPV: differential pulse voltammetry; EIS: electrochemical impedance spectroscopy.
